# Effectiveness of a culturally tailored HIV intervention in promoting PrEP among black women who use drugs in community supervision programs in New York City: a randomized clinical trial

**DOI:** 10.1186/s13722-024-00488-0

**Published:** 2024-07-23

**Authors:** Dawn Goddard-Eckrich, Tara McCrimmon, Keosha Bond, Mingway Chang, Timothy Hunt, Jennifer Hall, Mary Russo, Vineha Ramesh, Karen A. Johnson, Dget L. Downey, Elwin Wu, Nabila El-Bassel, Louisa Gilbert

**Affiliations:** 1 Social Intervention Group (SIG), Columbia University School of Social Work, 1255 Amsterdam Avenue, 8th Floor, New York, NY 10027 USA; 2 Sociomedical Sciences Department at the Mailman School of Public Health, 722 W. 168th Street, 16th floor, New York, NY 10032 USA; 3 School of Medicine, Community Health & Social Medicine, City University of New York, Harris Hall, H-313I, New York, NY 10031 USA; 4 University of Alabama School of Social Work, Box 870314, Tuscaloosa, AL 35487-0314 USA; 5 Silver School of Social Work, New York University, 1 Washington Square North, New York, NY 10003 USA

**Keywords:** PrEP, Community supervision, Drug use, Black women, Pre-exposure prophylaxiBackground

## Abstract

**Background:**

In the U.S. there are significant racial and gender disparities in the uptake of pre-exposure prophylaxis (PrEP). Black Americans represented 14% of PrEP users in 2022, but accounted for 42% of new HIV diagnoses in 2021 and in the South, Black people represented 48% of new HIV diagnoses in 2021 but only 21% of PrEP users in 2022. Women who use drugs may be even less likely than women who do not use drugs have initiated PrEP. Moreover, women involved in community supervision programs (CSP) are less likely to initiate or use PrEP, More PrEP interventions that focus on Black women with recent history of drug use in CSPs are needed to reduce inequities in PrEP uptake.

**Methods:**

We conducted a secondary analysis from a randomized clinical trial with a sub-sample (*n* = 336) of the total (*N* = 352) participants from the parent study (E-WORTH), who tested HIV negative at baseline were considered PrEP-eligible. Black women were recruited from CSPs in New York City (NYC), with recent substance use. Participants were randomized to either E-WORTH (*n* = 172) an HIV testing plus, receive a 5-session, culturally-tailored, group-based HIV prevention intervention, versus an HIV testing control group (*n* = 180). The 5 sessions included an introduction to PrEP and access. This paper reports outcomes on improved awareness of PrEP, willingness to use PrEP, and PrEP uptake over the 12-month follow-up period. HIV outcomes are reported in a previous paper.

**Results:**

Compared to control participants, participants in this study assigned to E-WORTH had significantly greater odds of being aware of PrEP as a biomedical HIV prevention strategy (OR = 3.25, 95% CI = 1.64–6.46, *p* = 0.001), and indicated a greater willingness to use PrEP as an HIV prevention method (b = 0.19, 95% CI = 0.06–0.32, *p* = 0.004) over the entire 12-month follow-up period.

**Conclusions:**

These findings underscore the effectiveness of a culturally-tailored intervention for Black women in CSP settings in increasing awareness, and intention to initiate PrEP. Low uptake of PrEP in both arms highlight the need for providing more robust PrEP-on-demand strategies that are integrated into other services such as substance abuse treatment.

**Trial Registration:**

ClinicalTrials.gov Identifier: *NCT02391233*.

**Supplementary Information:**

The online version contains supplementary material available at 10.1186/s13722-024-00488-0.

## Background

Increasing pre-exposure prophylaxis (PrEP) usage among historically **underreached** populations like Black women, individuals who use substances, and populations in the criminal legal system is consistent with U.S.’ Ending the HIV Epidemic in the U.S. (EHE) initiative, which focuses on scaling up science-based strategies that aim to end the HIV epidemic through strategic priorities [1–4] Black women are disproportionately impacted by the HIV epidemic in the United States (US), comprising 54% of new diagnoses among women, despite being only 13% of the female population in 2020, and with 10.9 times the rate of new HIV infections as non-Hispanic white women [5, 6]. Substance abuse amplifies HIV risks [7, 8] and longstanding racialized drug laws and policing in the US have resulted in a concentration of Black women in the criminal legal system [9]. This includes community supervision programs (CSPs, including probation, parole, and alternative-to-incarceration programs), the largest segment of the criminal legal system [10–12]. Among Black women in CSPs, hazardous drinking and substance use disorders (SUDs) range as high as 70% [13]. At the same time, these women are less likely than white women to access substance use treatment, and HIV prevention and treatment [13, 14]. HIV prevalence among persons engaged in the criminal legal system is about five times greater than in the general population, driven by poverty, unstable housing, lack of access to health care and insurance, and social networks composed of other high-risk individuals [15–17]. As expected, studies have shown high prevalence rates of HIV (17%). among samples of women in CSPs; among one sample of 337 women in CSPs in New York City, Black women accounted for 82% of all HIV-positive cases [16, 18] but less than 2% of Black women are prescribed PrEP [19].

Despite these risks, there is a lack of HIV preventive programming and services tailored to the needs of this population, including PrEP uptake. PrEP is an effective biomedical method of HIV prevention. However, Black women are four times less likely to have been prescribed PrEP than their non-Hispanic white female counterparts [20]. Individual and structural barriers may limit PrEP uptake among Black women in the US. Awareness of PrEP is notably low among Black cisgender women who have sex with men, with only 14% reporting PrEP awareness, compared to 21% of white cisgender women [21]. The targeted social marketing of PrEP has often emphasized the sexual behaviors of people who use PrEP (i.e., men who have sex with men) and sexual partner characteristics such as injection drug use or HIV status, contributing to the underestimation of risk among cisgender women more broadly [22–24]. Higher PrEP-related stigma has been shown to be associated with lower intention of initiating PrEP among Black and Latina urban women^17^. Medical mistrust and systemic racism may also operate as mediating mechanisms in the relationship between race and PrEP uptake, disproportionately affecting low-income Black women by reducing their access to preventative services and information [10, 25, 26]. Medical mistrust has been shown to be also negatively correlated with discussions of HIV prevention with healthcare providers [27–29].

There are few existing studies on PrEP among women in the criminal legal system. Low perceived risk may prevent women from initiating PrEP during incarceration, and competing priorities and schedule conflicts may prevent women from initiating PrEP when they return to the community, detrimentally impacting healthcare decision-making and the overall health outcomes of already marginalized populations [30]. One study conducted among women involved in the criminal legal system showed that 33% of women met the eligibility criteria for PrEP, but only 25% were aware of PrEP and only 16.7% of those who were eligible for PrEP perceived themselves as at risk for HIV [29]. No estimates are available of actual PrEP uptake among incarcerated or otherwise criminal legal-involved female populations.

Most U.S.-based PrEP interventions target men who have sex with men (MSM) [5] in urban areas and have leveraged information dissemination through social networks and peer leaders. Social media-based approaches have likewise shown promise in reaching high-risk, hard-to-reach participants [31, 32]. While interventions focusing on women’s PrEP uptake are in development, only few have targeted Black women, women with recent substance use, or women under community supervision [33, 34]. This represents a critical gap, as interventions that directly address the effects of racism and heterosexism are successful in improving the participation of underserved minoritized groups in the HIV care continuum [35]. Strategies that concomitantly aim to improve knowledge about PrEP while addressing associated social barriers, including racism and medical mistrust, may be key to its effective scale-up [36, 37]. Effective HIV prevention intervention and PrEP initiation must acknowledge and accommodate the specific needs and experiences of Black women who use substances under community supervision.

In response to this identified gap, this paper presents the results from the study Empowering African-American Women on the Road To Health, or E-WORTH. E-WORTH was a randomized controlled trial addressing HIV prevention among Black women with recent substance use under community supervision. This study was conducted from 2015 to 2018 in New York City. Secondary outcomes included reducing drug use, increasing use of drug treatment, linkage to HIV care and antiretroviral therapy adherence (for HIV-positive participants), and decreasing the incidence of IPV and recidivism. Results on primary outcomes showed that compared to the control group, participants in the E-WORTH intervention had 54% lower odds of testing positive for any STI at the 12-month follow-up. They also reported 38% fewer total acts of condomless sex and 42% fewer condomless sex acts with their main partners over the 12-month period. E-WORTH participants were more likely to consistently use condoms with all partners and their main partners. The intervention was effective in reducing STI incidence and increasing condom use among Black women, demonstrating the benefits of a culturally tailored approach. More details from the full study have been reported elsewhere [13, 38, 39]. The aim of this paper is to evaluate the effectiveness of the E-WORTH intervention on the secondary outcomes of knowledge about PrEP, intentions regarding use of PrEP, and actual use of PrEP at baseline to 3-, 6-, and 12-months post-intervention, as well as over the entire 12-month follow-up period.

## Methods

Full protocols for the parent study E-WORTH have been described elsewhere [38]. All procedures received approval from the Institutional Review Board at Columbia University.

### Participants and recruitment

**Fig. 1:**
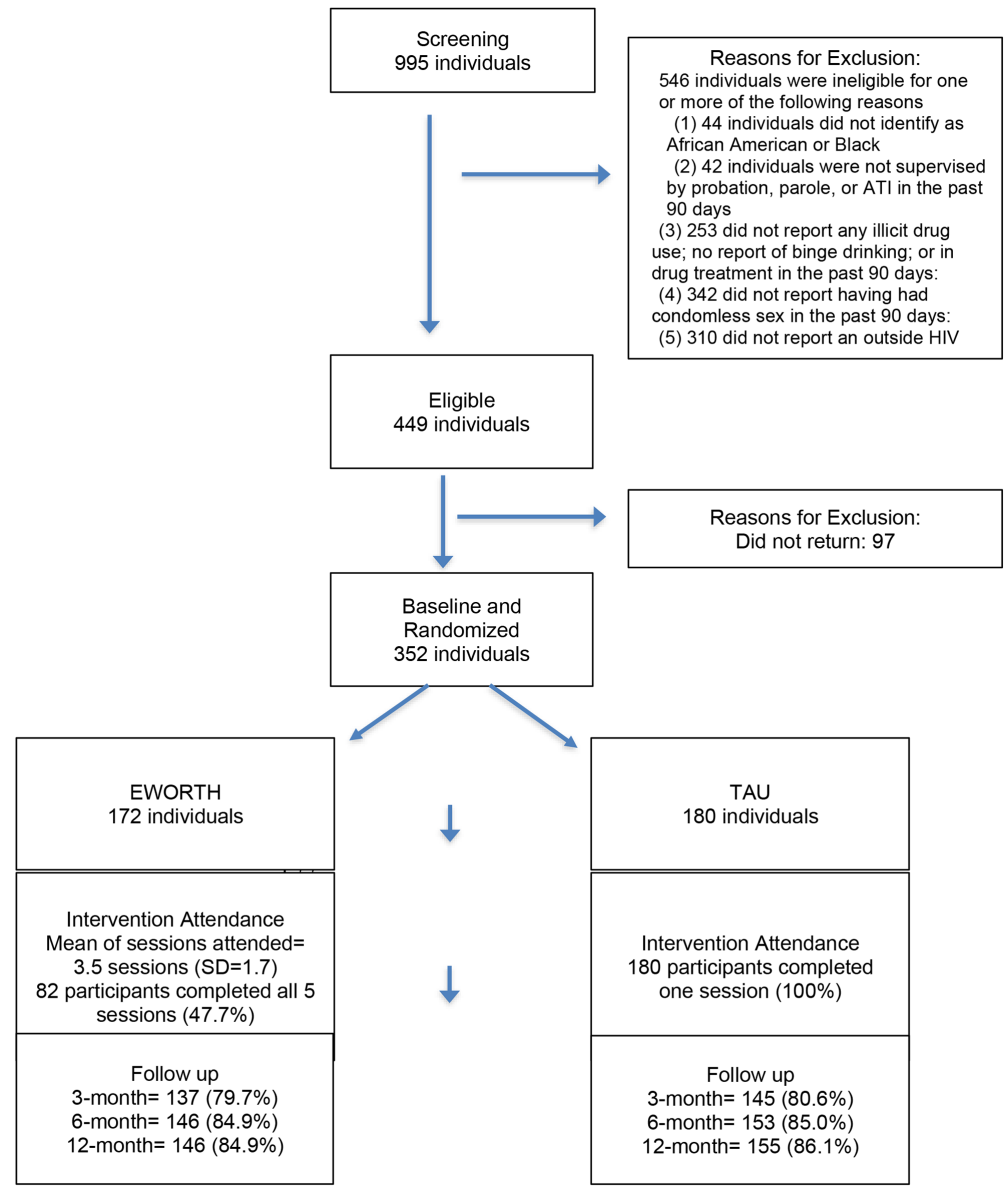
CONSORT form

For this study, we recruited women from community supervision programs (CSPs) in New York City. Probation, parole, and alternative-to-incarceration (ATI) programs were selected based on location and the number of Black women they serviced. We screened for eligibility using the following criteria: 1)18 years or older; 2) identification as African American/Black; 3) on probation, parole, or in an ATI program in the past 90 days; 4) any binge drinking or illicit drug use or enrollment in a drug treatment program in the past 90 days; 5) had condomless sex in the past 90 days; and 6) reported other HIV/STI risks in the past year (e.g., sex with multiple partners, syringe sharing) and/or being HIV positive). Our sub-sample included (*n* = 336) of the total (*N* = 352) participants from the parent study, who were eligible and were randomized to either a treatment-as-usual (TAU), the HIV testing control condition or the E-WORTH intervention condition (described below). Participants completed baseline assessments prior to randomization and were also assessed at three, six-, and twelve months post-intervention (measures described below). The analysis for this study excluded participants who were living with HIV at baseline (*n* = 16), resulting in a sample size of 336 women. Additional details regarding the methods have been described in the study’s main outcome paper [13]. Also see Fig. 1: CONSORT form.

### HIV testing control condition (treatment-as-Usual))

The HIV testing intervention was found to be effective in increasing oral HIV testing in drug treatment programs in the National Institute on Drug Abuse Clinical Trial Network 0032 HIV Testing study [40]. This took place in a single 30-minute session, comprised of: (1) viewing a 5-minute HIV rapid testing information video from OraSure [41, 42]; (2) reviewing the OraSure testing pamphlet on transmission risks and strategies for reducing risks; (3) taking the rapid OraQuick oral HIV test; and (4) receiving the test results and a service manual for PrEP resources/services and other ancillary services. Participants also had ongoing access to free condoms and safer sex kits at the CSP sites where they were recruited from.

### Intervention condition

The E-WORTH condition, informed by empowerment theory and social cognitive theory with a health equity lens, provided participants with individual, group, and hybrid experiences to enhance HIV/STI knowledge, skills, and self-efficacy to reduce associated risks [43–45]. Detailed descriptions of the intervention adaptation process, core elements and primary outcomes are available elsewhere [13, 46]. Intervention content was tailored to the specific needs of Black women with a recent history of substance use. E-WORTH consisted of five sessions: The first session was identical to the TAU HIV Testing Control Condition. Participants were also introduced to goal-setting and received referrals for ancillary services. Sessions 2–4 were weekly, 90-minute, in-person group sessions led by a Black female facilitator who guided opening and closing activities and introduced the individualized computerized interactive activities. The computerized intervention components used videos led by a Black female guide and additional Black female characters.

Core components of the video intervention included sessions that were a combination of individual and group sessions that focused on HIV/STI treatment and drug use and had discussions and activities that included: (1) Enhancing motivation and linkage to HIV/STI treatment and drug treatment, raised awareness of and set goals around HIV/STI drug use; (2) Negotiating and Managing Drug use through: introduce, model and role play negotiation skills for safer sex and drug use through simulated vignettes including how to managing sexual and drug risk using a problem solving (POP) techniques and identify drug-related triggers for unsafe sex using interactive exercises; (3) Revisiting a social support network map that identified people, places and things that were possible triggers for unsafe sex and drug use; (4) Identifying factors that can increase risk through drug and alcohol use and review strategies for safe injection practices; and (5) Developing a personalized safety plan [13, 38]. For E-WORTH PrEP-specific content, we provided detailed information and resources on PrEP and integrated it into HIV risk reduction goal-setting and provided service linkages for PrEP with the option to go to a PrEP approved clinics provided by the New York City Department of Health or to consult with their private doctor.

All participants received a comprehensive referral manual tailored for drug-using black women involved in the criminal legal system. HIV content focuses on consistent condom use and taking PrEP if not able to use a condom to prevent HIV infection; normalizing HIV/STI testing and if they contracted HIV, building commitment and skills to take medications to lower transmission risks to sexual and drug sharing partners.

### Measures

Participants completed computer-assisted self-interviewing at four time periods: baseline (prior to randomization), then 3-, 6-, and 12-month follow-up points post-intervention.

#### Sociodemographic and risk characteristics

Sociodemographic data was collected from participants in their baseline assessment. Participants were asked about their age, ethnicity, employment status, and marital status.

Substance use indicators included whether participants had participated in binge drinking, and illicit drug use, and whether they ever injected drugs both ever and in the past 30 days.

Sexual risk indicators included whether participants had more than one sexual partner, had exchanged sex for money or food, and the number of unprotected sex acts with all sexual partners. Participants were asked if they were diagnosed with an STI and trichomoniasis, chlamydia and gonorrhea, and a single dichotomous measure of testing positive for any STI was calculated. Additionally, women obtained a self-collected vaginal swab or urine sample during their baseline visit to test for *Trichomonas vaginalis*, *Chlamydia trachomatis*, and *Neisseria gonorrhoeae* and sent to the Bio Reference Laboratory.

#### Outcome measures

PrEP outcomes were measured using three variables. **PrEP Awareness** was measured using a dichotomous item. Participants were given a description of (PrEP **reduces** their risk of getting HIV from HIV positive partners) and then asked: *Have you heard of PrEP as an antiretroviral medication that you can take to reduce your risk of getting HIV from HIV positive partners?*

##### Willingness to use PrEP

was measured by a single question: *If PrEP were available to you*,* how likely would you be willing to use PrEP as an HIV prevention method*? Participants selected a response of 1 = very unlikely, 2 = unlikely, 3 = likely, 4 = very likely.

##### PrEP Uptake

was measured using a dichotomous question: *Have you taken Truvada or another antiretroviral medication to reduce your likelihood of getting HIV in the past 90 days?*

### Statistical analysis

Descriptive statistics were generated for all variables at the baseline and for the PrEP outcome measures each follow-up assessment by study condition. Baseline sociodemographics and behavioral factors were compared between the E-WORTH and TAU arms at baseline using two-tailed t-tests or Chi-squared tests to determine if any covariate adjustment was needed in the final model. To examine the effects of E-WORTH, we employed mixed-effects logistic regression for the awareness outcome of PrEP and mixed-effects linear regression for the intended outcomes of PrEP use. The mixed effects generalized linear models included a random effect for repeated measures and covariate adjustments for age, high school, employment, marital status, a positive test for any STI at baseline, and a baseline measure of the outcomes. To estimate the average effects at the entire follow-up period, treatment conditions along with the covariates were included in the models. To estimate the effects at each follow-up, we included the intervention condition, follow-up-up time, and interaction between the intervention condition and follow-up time and covariate adjustments in the models. We then obtained the effects at each follow-up by linear combinations of the parameters associated with the intervention condition and interaction between the intervention condition and respective follow-up time. Intervention effects were reported as regression coefficients (b) from mixed-effects linear regression and odds ratios (OR) from mixed-effects logistic regression models. Statistical significance was assessed using the associated 95% confidence interval (CI) and p-value for each estimate. This study also excluded 17 participants (one missing case, 16 HIV positive cases removed from the total study sample, owing to missing demographic data for a final sample size of 336. Statistical analyses were performed using Stata 15.

## Results

Analyses were conducted among a sub-sample of Black women (*n* = 336), under community supervision who tested negative for HIV at baseline. Table 1 presents the descriptive statistics for sociodemographic characteristics, substance use, and criminal legal involvement among the sample and differences by condition. The mean age was 31.8 years (SD = 10.7). Over one-fifth (*n* = 76, 22.7%) identified themselves as Latinx. Less than half were single and never married (*n* = 144, 41.5%). More than half completed high school or GED (*n* = 187, 55.8%), and less than one-third (*n* = 100, 29.9%) were employed. Over two-thirds reported lifetime binge drinking (*n* = 241, 69.5%), and 83.3% (*n* = 279) reported drug use in their lifetime. Most participants were on probation (*n* = 241, 71.9%), 16.4% (*n* = 55) were on parole, and 17.6% (*n* = 59) were in an ATI program in the previous 90 days. At baseline 31.5% tested positive for an STI (27.6% Trichomoniasis, 5.4% chlamydia, 1.8% Gonorrhea), 48.4% of women had more than one sexual partner, 15.8% exchanged sex for money or foods, and the women reported an average of 22.2 (SD = 30.9) unprotected sex acts across partners in the previous 90 days. Except for any STI positive, no other significant differences were found between conditions at baseline.

**Table 1:** Characteristics of the sample by study arm at the baseline assessment, EWORTH Effectiveness Trial, New York, 2015–2019 (*n* = 336)

	No. (%)		
	Total (*N* = 336)^a^	Control (*n* = 173)^a^	EWORTH (*n* = 163)
*Sociodemographic Indicators*			
Age, mean (SD)	31.8 (10.7)	31.6 (10.7)	32.1 (10.7)
Latinx ethnicity (All identified as Black during screening)	76 (22.7%)	42 (24.4%)	34 (20.9%)
Married, including common-law marriage	58 (17.3%)	25 (14.5%)	33 (20.2%)
High school graduate/GED	187 (55.8%)	96 (55.8%)	91 (55.8%)
Employed	100 (29.9%)	55 (32.0%)	45 (27.6%)
*Substance Use Indicators*			
Ever binge drinking	232 (69.3%)	122 (70.9%)	110 (67.5%)
Binge drinking in the past 30 days	146 (43.6%)	75 (43.6%)	71 (43.6%)
Used any illicit drug in the past 30 days	198 (59.1%)	102 (59.3%)	96 (58.9%)
Ever injected drugs	14 (4.2%)	5 (2.9%)	9 (5.5%)
Injected drugs in the past 90 days	6 (1.8%)	3 (1.7%)	3 (1.8%)
*Sexual risk indicators*			
Tested positive for an STI ^b^	105 (31.5%)	44 (25.6%)*	61 (37.9%)*
More than one sexual partner in the past 90 days	162 (48.4%)	84 (48.8%)	78 (47.9%)
Exchange sex for money/food in the past 90 days	53 (15.8%)	26 (15.1%)	27 (16.6%)
# of unprotected sex acts across partners in the past 90 days	22.2 (30.9)	24.8 (33.5)	19.5 (27.7)

* *p* < 0.05, ** *p* < 0.01 by two-tailed t-test or Chi-squared test between two **arms**; (a) One missing case, 16 HIV positive cases removed from the total study sample; (b) Three participants did not test for STIs

Table 2 presents descriptive statistics for the PrEP-related outcome measures at the baseline and each follow-up assessment by intervention assignment. Although some differences in PrEP relative measures between E-WORTH and control conditions were found at different follow-up assessments in Table 2, we reported the hypothesis testing for the intervention effects in Table 3. Compared to control participants, E-WORTH participants had significantly greater odds of being aware of PrEP as a biomedical HIV prevention strategy over the 12-month follow-up period (OR = 3.25, 95% CI = 1.64–6.46, *p* = 0.001), and indicated a significantly higher willingness to use PrEP as an HIV prevention method (b = 0.19, 95% CI = 0.06–0.32, *p* = 0.004).

**Table 2:** PrEP related measures at the baseline and each follow-up among HIV negative participants: # (%) or mean (SD) (*n* = 336)

	Arm	Baseline (*n* = 336)	3-month (*n* = 272)	6-month (*n* = 285)	12-month (*n* = 288)
**PrEP Awareness**					
Have you *heard of PrEP* as an antiretroviral medication that you can take to reduce your risk of getting HIV from HIV positive partners? (Yes/No)	TAU	43 (25.0%)	39** (27.7%)	48** (32.4%)	78** (53.1%)
	EWORTH	49 (30.1%)	73** (55.7%)	72** (52.6%)	98** (71.5%)
**Willingness to use PrEP**					
If PrEP is available to you, how likely would you be *willing to use PrEP* as an HIV prevention method? (Range: 1–4)	TAU	2.52 (1.16)	2.39* (1.15)	2.45 (1.08)	2.28 (1.14)
	EWORTH	2.58 (1.19)	2.70* (1.14)	2.47 (1.24)	2.50 (1.18)
**PrEP Uptake**					
Have you *taken Truvada or another antiretroviral medication* to reduce your likelihood of getting HIV in the past 90 days? (Yes/No)	TAU	1 (0.7%)	2 (1.5%)	2 (1.4%)	4 (2.7%)
	EWORTH	0 (0%)	0 (0%)	2 (1.5%)	3 (2.2%)

* *p* < 0.05, ** *p* < 0.01 by two-tailed t-test or Chi-squared test between two arm

**Table 3:** Results from mixed effects models for intervention effect estimates at each follow-up and over the entire follow-up period: adjusted, bootstrapping (2000 replications), HIV negative sample (*n* = 336)

	Entire follow-up	3-month	6-month	12-month
**PrEP Awareness**				
Have you *heard of PrEP* as an antiretroviral medication that you can take to reduce your risk of getting HIV from HIV positive partners? (*OR*)	3.25** [1.64,** 6.46] (***p*** = 0.001)**	4.66* [1.31,** 16.55] (***p*** = 0.017)**	3.98** [1.41,** 11.26] (***p*** = 0.009)**	2.89 [0.79, 10.59] (*p* = 0.108)
**Willingness to use PrEP**				
If PrEP is available to you, how likely would you be *willing to use PrEP* as an HIV prevention method? (*b*)	0.19** [0.06,** 0.32] (***p*** = 0.004)**	0.20 [-0.002, 0.403] (*p* = 0.053)	0.19** [0.06,** 0.33] (***p*** = 0.006)**	0.18 [-0.04, 0.40] (*p* = 0.116)

* *p* < 0.05, ** *p* < 0.01; Note: Covariate adjustments: age, high school, employed, married, STI at baseline and baseline measure of the outcomes

## Discussion

This study examined the efficacy of the E-WORTH intervention in increasing PrEP awareness and willingness to use PrEP among Black women under community supervision who were HIV-negative at baseline. The results demonstrated that participants in the E-WORTH condition had significantly greater odds of being aware of PrEP as an HIV prevention strategy and reported higher willingness to use PrEP compared to the control group over the 12-month follow-up period. Our findings indicate that compared to the control group, the E-WORTH group had significantly greater odds of being aware of PrEP as a biomedical HIV prevention strategy. **Our findings** also **show** that Black women who use substances in CSPs had a significantly higher willingness to use PrEP and also reported a greater willingness to use condoms while taking PrEP over the entire 12-month follow-up assessment. These findings suggest that the E-WORTH intervention was effective in increasing awareness and willingness to use PrEP among the participants, known to be strong predictors of behavior and have implications for scaling up public sector PrEP among women involved in the criminal legal system.

PrEP awareness among Black women in this study is similar to other studies with community samples of Black women [12, 47]. While there is a wide body of literature that includes women with substance use disorder, this is one of the few PrEP studies with cisgender women that document awareness of, intentions regarding, and actual use of PrEP among Black women who use substances. A general lack of information about PrEP and a limited ability to appropriately identify their degree of exposure, even among women with an increased likelihood of acquiring HIV, appear to be major contributors to PrEP underutilization among U.S. women [22–24]. However, our study sheds light on the awareness, intentions and usage of PrEP, among women who use substances. It’s crucial to interpret our results in the context of substance use disorder (SUD) and how it impacts PrEP outcomes. Previous studies have delved into this connection especially concerning women with SUD highlighting both obstacles and support for adoption and adherence. Our findings align with and diverge from existing research emphasizing the intricate interplay between substance use sociodemographic factors and behaviors related to PrEP. Future studies should delve deeper into how cultural, contextual and structural factors might mediate or moderate the link between SUD and PrEP outcomes, in this under researched group.

Not unexpectedly, awareness of PrEP was low among Black women in a CSP setting where there have been minimal efforts to disseminate information about or provide free access to PrEP. A multi-stage and culturally tailored, targeted education campaign will be needed to increase PrEP literacy and generate demand. Also, the lack of knowledge about HIV and underestimation of personal HIV susceptibility remains barriers to the uptake of PrEP and other HIV prevention interventions. In this regard, bundling PrEP with other services (e.g., drug use treatment, sexually transmitted infection services, reproductive services) may expedite linking individuals who experience multiple factors that contribute to HIV transmission to PrEP.

This finding that Black women who received the E-WORTH condition are also more willing to use condoms while taking PrEP also affirms the potential for STI and pregnancy prevention to Black women thus further interventions that address PrEP should include condom use and information on STI’s and pregnancy prevention. Previous research with Black women have raised additional worries regarding how PrEP will actually fit into women’s life in terms of gendered social and economic costs [48]. The other significant barrier is that the social marketing of PrEP is almost entirely towards MSM, inadvertently reinforcing stigma towards this population while alienating other populations at high risk for HIV [32, 49]. Moreover, the potential long-term implications of PrEP on women’s reproductive health have been identified as a barrier to initiation, but injectable PrEP might reduce these adherence barriers [50]. These findings emphasize the significance of a contextualized and integrated approach to women’s sexual and reproductive health care. Further, the sample of participants experienced multiple factors that contributed to their vulnerability to HIV transmission.

The sample of participants experienced multiple factors that contributed to their vulnerability to HIV transmission, including high poverty indicators, are prevalent among the sample population, with many reporting food insecurity and homelessness [51]. The results are consistent with previous research that investigates how poverty, including homelessness, neighborhood decline, housing issues, and a general lack of social capital in geographically defined areas, may contribute to HIV transmission and lack of awareness and uptake of PrEP as an effective prevention strategy [52, 53]. There were no significant differences between conditions on these variables, indicating that these factors were equally prevalent across groups.

These results are aligned with other research on Black women involved in the criminal legal system who also experience l structural and systemic racism, discrimination, medical mistrust and medical racism as barriers to promoting PrEP among Black women [25, 33]. Finally, our findings also indicate that addressing specific individual-level barriers that may create opportunities for empowerment and advocacy among Black women can increase their knowledge, self-efficacy, and intentions to initiate PrEP and consistently use condoms for HIV prevention. The promising findings on the effectiveness of E-WORTH on increasing awareness and willingness to use PrEP as secondary outcomes, adding to the robust primary outcomes of the effectiveness of E-WORTH reducing STIs and unprotected sex among Black women in CSPs. Taken together, the findings suggest that E-WORTH is an effective HIV prevention strategy. Upgrading E-WORTH’s content to include a PrEP on-demand component and more activities to identify and reduce barriers to PrEP use might be needed to increase the uptake of PrEP. These findings suggest the need for targeted interventions to address the complex needs of Black women in CSP.

### Limitations

Implications need to be considered with light of limitations in this study. Our sample involved Black women in CSPs in New York City. As such, the findings are not generalizable to the broader population of Black women who are not in CSPs yet also remain at increased risk of HIV acquisition and in need of responsive interventions that increase awareness and willingness to uptake PrEP. This study focused on individual participant awareness of and willingness to use PrEP with the larger goal of eventual increased uptake which could lower the prevalence of disproportionate HIV acquisition. While this data is helpful in designing targeted interventions, individual perspectives exist within larger social structures and systems. The study would have benefited from the application of an analytical framework that accounts for systemic obstacles, such as structural racism, in addition to individual perceptions. Finally, the regression coefficients is a limitation of the study as we relied on one single item for measuring willingness with assigned numeric 1–4 to the unlikely-likely responses. However, although the coefficients are not easily to be interpreted numerically, the coefficients indicate clearly that the EWORTH group reported higher willingness to use PrEP than the control group.

Despite these limitations, our study and sample have important implications for HIV prevention among racial/ethnic minoritized women and emphasizes the need to continue to expand HIV prevention strategies, including PrEP among women in CSP’s. It also further underscores the need to ensure that PrEP information is culturally tailored for Black women. Furthermore, since a large portion of the sample is also identified as Latinix, it is also important to culturally tailor to different types of Black women. Recent data has shown that the majority of African Americans are second generation and beyond immigrants, so these cultural norms and stigma may have further implications for future interventions [54]. Additionally, future research should explore a mixed methods methodology which would allow for qualitative analysis to explain the disconnection among participants between increased awareness of PrEP and continued low uptake.

## Conclusions

These findings suggest the effectiveness of a culturally-tailored intervention for Black women in CSP settings in increasing awareness, willingness, and intention to initiate PrEP. The low uptake of PrEP in both arms may be due to lack of access, but also may highlight the need for providing more robust PrEP-on-demand strategies (e.g., PrEP telemedicine) during the intervention rather than linkage to a PrEP provider. Advancing an effective PrEP intervention for Black women in CSPs holds great promise for reducing inequities in PrEP uptake and increasing HIV prevention among this underserved group. While our results do not directly address ways to increase awareness, uptake and sustained use of PrEP, increasing awareness and interest of PrEP alone is insufficient to drive behavior change and sustained use. To bridge the gap between awareness/interest and actual uptake and adherence, a multi-pronged approach is typically needed that include ensuring affordable and seamless integration of PrEP into healthcare systems, offering counseling and support to address concerns and encourage adherence, leveraging networks and influential figures for outreach, tailoring messages to cultural backgrounds, exploring innovative delivery methods like telemedicine and home delivery, and continuously monitoring and adjusting strategies. Thus, a comprehensive approach addressing individual, social, and structural factors, coupled with ongoing support, is necessary to bridge the gap between interest and consistent PrEP utilization.

### Electronic supplementary material

Below is the link to the electronic supplementary material.

Supplementary Material 1

Supplementary Material 2

Supplementary Material 3

## Data Availability

The data collected and analyzed for the current study are available from the corresponding author on reasonable request.

## Abbreviations

ATI: Alternative to Incarceration
CSP: Community supervision programmes
CLS: Criminal legal system
EHE: Ending the HIV Epidemic in the U.S
EWORTH: Empowering African-American Women on the Road To Health
MSM: Men who have sex with men
PrEP: Pre-exposure prophylaxis
SUD: Substance use disorder
STI: Sexual Transmitted Infection
TAU: Treatment as Usual
