# Supplementary material for: Effectiveness of a culturally tailored HIV intervention in promoting PrEP among black women who use drugs in community supervision programs in New York City: a randomized clinical trial
Source: Addict Sci Clin Pract. 2024 Jul 23;19:55. doi: 10.1186/s13722-024-00488-0 (PMC11264441; doi:10.1186/s13722-024-00488-0)
Supplement: Supplementary file 1 — Supplementary Material 1 [file 13722_2024_488_MOESM1_ESM.docx]

A**ngela Bazzi PhD**,
**Tyler Bartholomew PhD,**
**Hansel Tookes** PhD,

Guest Editors- Special Issue

[Addiction Science & Clinical Practice](https://ascpjournal.biomedcentral.com/) 

June 15, 2024, 2024

# RE: Special Issue: Pre-exposure Prophylaxis for People Who Use Drugs

Dear Drs. **Bazzi, Bartholomew and Tookes,**

I would like to resubmit the manuscript entitled “Effectiveness of a culturally tailored HIV intervention in promoting PrEP among Black women who use drugs, in community supervision programs in New York City: A randomized clinical trial to be considered for publication in your Special Issue on Pre-exposure Prophylaxis for People Who Use Drugs in [Addiction Science & Clinical Practice](https://ascpjournal.biomedcentral.com/). Also, our apologies for the delayed response to the reviewers.

We would like to thank you for your thoughtful and thorough critique, including your positive feedback on our manuscript. We have carefully considered each comment and used the feedback to make major revisions to our manuscript accordingly. As a result, we believe our revised manuscript has significantly improved and is much stronger than the original submission.

We have carefully re-evaluated the Discussion section to ensure the conclusions do not overreach beyond the data presented in the Results. Specifically:

- We have removed speculative statements about potential reasons for low PrEP uptake that were not directly supported by our results;
- We have reframed the discussion to focus on interpreting the findings from our study sample, rather than generalizing broadly;
- We have added caveats about the limitations of our study.

Regarding the low PrEP uptake observed in both conditions, although our study does not identify causal factors definitively, we propose several potential explanations based on further research and previous literature. We do hope that the revisions have strengthened our manuscript. Responses to the reviewer are ***italicized and BOLD*** in a separate attachment. Changes within the manuscript are tracked changes and highlighted in yellow.

As mentioned before, as Corresponding Author, I confirm that all authors listed have contributed sufficiently, approved this manuscript for submission, agree to the submission guidelines, and do not have any competing interests. We declare that this manuscript is original and is not currently under review elsewhere. We know of no conflicts of interest associated with this publication, and there has been no significant financial support for this work that could have influenced the outcome.

Thank you for your consideration.

Sincerely,


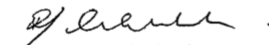


Dr. Dawn Goddard-Eckrich, EdD, MSS

Associate Director, Social Intervention Group,

Associate Research Scientist, Columbia University, School of Social Work,

New York, NY 10027
ph: [212-851-5660](tel:(212)%20851-5660); email: [dg2121@columbia.edu](mailto:dg2121@columbia.edu)
