# Supplementary material for: Effectiveness of a culturally tailored HIV intervention in promoting PrEP among black women who use drugs in community supervision programs in New York City: a randomized clinical trial
Source: Addict Sci Clin Pract. 2024 Jul 23;19:55. doi: 10.1186/s13722-024-00488-0 (PMC11264441; doi:10.1186/s13722-024-00488-0)
Supplement: Supplementary file 2 — Supplementary Material 2 [file 13722_2024_488_MOESM2_ESM.docx]

**Effectiveness of a culturally tailored HIV intervention in promoting PrEP among Black women who use drugs in community supervision programs in New York City: A randomized clinical trial**

Dawn Goddard-Eckrich^1^*, Tara McCrimmon^2^, Keosha Bond^3^, Mingway Chang^1^, Timothy Hunt ^1^, Jennifer Hall^1^, Mary Russo^1^, Vineha Ramesh^1^, Karen A. Johnson^4^, Dget Downey^1^, Elwin Wu^1^, Nabila El-Bassel^1^ and. Louisa Gilbert^1^

^1^Social Intervention Group (SIG), Columbia University, School of Social Work, 1255 Amsterdam Avenue, 8^th^ Floor, New York, NY 10027.

^2^Sociomedical Sciences Department at the Mailman School of Public Health., 722 W. 168th Street, 16th floor, New York, NY 10032, USA.

^3^City University of New York, School of Medicine, Community Health & Social Medicine, Harris Hall, H-313I, New York, NY 10031, USA

^4^University of Alabama School of Social Work, Box 870314. Tuscaloosa, AL 35487-0314, USA

*Corresponding author: Dawn Goddard-Eckrich, EdD, Social Intervention Group, Columbia University School of Social Work, 1255 Amsterdam Avenue, Room 802, New York, NY. Phone: 212-851-2395; Email: [dg2121@columbia.edu](mailto:dg2121@columbia.edu); Fax: 212-851-2126; Linkedin: <https://www.linkedin.com/in/dr-dawn-goddard-eckrich-edd-mss-aaa6556b/>
