# Supplementary material for: Effectiveness of a culturally tailored HIV intervention in promoting PrEP among black women who use drugs in community supervision programs in New York City: a randomized clinical trial
Source: Addict Sci Clin Pract. 2024 Jul 23;19:55. doi: 10.1186/s13722-024-00488-0 (PMC11264441; doi:10.1186/s13722-024-00488-0)
Supplement: Supplementary file 3 — Supplementary Material 3 [file 13722_2024_488_MOESM3_ESM.docx]

**ASCP-D-23-00120**

**Effectiveness of a culturally tailored HIV intervention in promoting PrEP among Black women who use drugs in community supervision programs in New York City: A randomized clinical trial.**

**Addiction Science & Clinical Practice**

**Editor's comments:**

Two expert reviewers and I read this paper with interest. We are enthusiastic about the topic and sample yet share some concerns about areas of the manuscript that could be improved. In addition to addressing each of the reviewers’ comments (noting that some comments are multi-part), please pay particular attention to ensuring that the conclusions (in the Discussion section) do not reach beyond the results reported (i.e., that the discussion points are fully supported by data presented in the Results section, as noted by R1), and to the excellent questions posed by R2 at the end of their review about why there was such low PrEP uptake (which also somewhat echo R1’s comments). Addressing these concerns and elevating the discussion and conclusion points around reasons for low uptake will help make this work more impactful and a stronger contribution to the literature.

**Response to Editor:**

We would like to thank you for your thoughtful and thorough critique, including your positive feedback on our manuscript. We have carefully considered each comment and used the feedback to make major revisions to our manuscript accordingly. As a result, we believe our revised manuscript has significantly improved and is much stronger than the original submission.

We have carefully re-evaluated the Discussion section to ensure the conclusions do not overreach beyond the data presented in the Results. Specifically:

- We have removed speculative statements about potential reasons for low PrEP uptake that were not directly supported by our results;
- We have reframed the discussion to focus on interpreting the findings from our study sample, rather than generalizing broadly;
- We have added caveats about the limitations of our study.

Regarding the low PrEP uptake observed in both conditions, although our study does not identify causal factors definitively, we propose several potential explanations based on further research and previous literature.

We hope the revisions have strengthened our manuscript. Responses to the reviewer are ***italicized BOLD*** below. Changes within the manuscript are tracked changes and highlighted in yellow.

Thank you!

**ASCP-D-23-00120**

**Effectiveness of a culturally tailored HIV intervention in promoting PrEP among Black women who use drugs in community supervision programs in New York City: A randomized clinical trial.**

**Reviewer #1:** The authors present a post hoc (I assume) analysis of outcomes of PrEP awareness, willingness by self-report to start and actual uptake from a completed study. The authors are to be commended for exploring these outcomes in this population of Black women in CSP. However, the study has some limitations in the data, analysis and discussion which are discussed below.

**Response to Reviewer #1**

***We would like to thank the reviewer for their thoughtful comments and suggestions to improve our manuscript. We appreciate the reviewer acknowledging the importance of exploring PrEP awareness, willingness, and uptake in this understudied population of Black women in the criminal-legal system. Regarding the limitations noted by the reviewer, we agree that our study has some limitations which we have aimed to transparently discuss in the manuscript. We have reviewed all of your comments and suggestions and have updated all of the sections to make this easier to understand. Please see our responses in BOLD below.***

***Thank you.***

_____________________________________________________________________________________________________________________

**(Reviewer 1, Abstract)**

1. In the abstract, the methods mention increased uptake, but those data are not reported.

***Thank you for your comment. We have now reworded the abstract background and included data to accurately support our statement.***

*“Black Americans represented 14% of PrEP users in 2022, but accounted for 42% of new HIV diagnoses in 2021 and in the South, Black people represented 48% of new HIV diagnoses in 2021 but only 21% of PrEP users in 2022.”*

2. In addition, while one data point showed higher willingness (and I assume drive the mixed effects model results), this was not seen in follow-up time periods, even if remained significant across the study period.

***Although the willingness was only significant at 6 months at the 0.05 level, the 3-months effect was very close to significant (p=0.053). Thus, the significance for the entire period is not only driven by one data point.***

**(Reviewer 1, Introduction)**

3. The statement "While interventions focusing on women's PrEP uptake are in development, none target Black women, women with recent substance use, or women under community supervision [33,34]." Needs updating as there are interventions in development, just results have not been published. (ex. <https://doi.org/10.1016/j.whi.2023.11.001> which may have come out after submission)

***We submitted this manuscript to the journal in September 2023, so this recently published paper you included was not yet published. We have updated this paragraph and included the published paper in our references.***

***This has been added to references:***

*Wyatt GE, Norwood-Scott E, Cooley-Strickland M, Zhang M, Smith-Clapham A, Jordan W, Liu H, Hamilton AB. Increasing Urban African American Women's Readiness for Pre-exposure Prophylaxis: A Pilot Study of the Women Prepping for PrEp Plus Program (WP3+). Womens Health Issues. 2024 May-Jun;34(3):241-249. doi: 10.1016/j.whi.2023.11.001. Epub 2024 Jan 23. PMID: 38267337.*

4. While the authors note that primary outcomes were already reported, including a brief description in the introduction would be important as not all readers will go to the primary paper and helps understand associated change (or not) in these outcomes

***We have included a brief paragraph on the primary outcomes of the parent study.***

*Results on primary outcomes showed that compared to the control group, participants in the E-WORTH intervention had 54% lower odds of testing positive for any STI at the 12-month follow-up. They also reported 38% fewer total acts of condomless sex and 42% fewer condomless sex acts with their main partners over the 12-month period. E-WORTH participants were more likely to consistently use condoms with all partners and their main partners. The intervention was effective in reducing STI incidence and increasing condom use among Black women, demonstrating the benefits of a culturally tailored approach. More details from the full study have been reported in another journal [13,38,39].*

**(Reviewer 1, Methods)**

5. The authors need to describe how the PrEP willingness question was chosen and any validation in this population.

***Our investigative team designed this question for the PrEP questions for a prior NIH study we conducted. However, those study PrEP results are currently in draft and have not been published. Additionally, we think this willingness question is a very standard and straightforward question that may have been used in other studies. or we can't the source, can we use the same rationale using qualitative interviews as the response to the awareness question?***

6. Why the PrEP awareness question was asked after "PrEP Participants were given a description of (PrEP reduce their risk of getting HIV from HIV positive partners) "

***Based on qualitative interviews we conducted with a prior study with persons involved in the criminal legal system, many participants had heard about PrEP but did not remember the name of it. Therefore, the rationale for providing a description on what PrEP stands for was to ensure that participants understood what the acronym PrEP refers to so that we could assess their actual level of prior knowledge of PrEP rather than a lack of understanding about what the acronym PrEP refers to. This approach increases the validity and interpretability of the PrEP awareness responses. We have therefore added these to the discussion with appropriate citations.***

**(Reviewer 1, Discussion)**

7. The discussion is well written in style but draws conclusion that as a reader I found hard to map from the findings presented. Some examples are below, but overall, the broader statements (while correct), are not necessarily supported by the finding.

***Thank you for your positive feedback. We have reviewed your selected statements and have toned down some of our statements to support our findings. We have carefully re-evaluated the Discussion section to ensure the conclusions do not overreach beyond the data presented in the Results. Specifically:***

- ***We have provided an explanation about potential reasons for low PrEP uptake that were not directly supported by our results.***
- ***We have reframed the discussion to focus on interpreting the findings from our study sample, rather than generalizing broadly.***
- ***We have added caveats about the limitations of our study.***

8. The paragraph about financial instability. In addition, the discussion should start with your findings and then reflect on their relevance

***We have reorganized the beginning of the discussion to review findings and then reflect on the relevance. We removed the financial instability and other pieces that gave broad statements. Here is the new first paragraph in the discussion section:***

*“This study examined the efficacy of the E-WORTH intervention in increasing PrEP awareness and willingness to use PrEP among Black women under community supervision who were HIV-negative at baseline. The results demonstrated that participants in the E-WORTH condition had significantly greater odds of being aware of PrEP as an HIV prevention strategy and reported higher willingness to use PrEP compared to the control group over the 12-month follow-up period. While there is a wide body of literature that includes women with substance use disorder, this is one of the few PrEP studies with cisgender women that document awareness of, intentions regarding, and actual use of PrEP among Black women who use substances. Our findings indicate that compared to the control group, the E-WORTH group had significantly greater odds of being aware of PrEP as a biomedical HIV prevention strategy. It also shows that Black women who use substances in CSPs had a significantly higher willingness to use PrEP and also reported a greater willingness to use condoms while taking PrEP over the entire 12-month follow-up assessment. These findings suggest that the E-WORTH intervention was effective in increasing awareness and willingness to use PrEP among the participants, known to be strong predictors of behavior.”*

9. As noted above, the statement, that "This is one of the few PrEP studies with cisgender women that document awareness of intentions regarding, and actual use of PrEP among Black women who use substances." While in part true, neglects the deep literature which includes women with substance use disorder which should be noted and results from these papers when available to look at relationship of SU and the outcomes of awareness and willingness as well as uptake compared.

***Thank you for highlighting the need to acknowledge the broader SUD literature. We will summarize key findings exploring SUD's relationship with PrEP outcomes and discuss how our results align/diverge, offering explanations. We will also situate our focus on understudied Black women who use substances within this context. Additionally, we will note limitations regarding the SUD literature and suggest future research directions examining potential mediators/moderators influencing substance use and PrEP factors. We think that incorporating these elements will strengthen our positioning and impact. We have added the following paragraph to our discussion section:***

“Our study sheds light on the awareness, intentions and usage of PrEP, among women who use substances. It's crucial to interpret our results in the context of substance use disorder (SUD) and how it impacts PrEP outcomes. Previous studies have delved into this connection especially concerning women with SUD highlighting both obstacles and support for adoption and adherence. Our findings align with and diverge from existing research emphasizing the intricate interplay between substance use sociodemographic factors and behaviors related to PrEP. Future studies should delve deeper into how cultural, contextual and structural factors might mediate or moderate the link between SUD and PrEP outcomes, in this under-researched group.”

10. The higher willingness to use condoms while on PREP is not in the results table and should be included in describing the primary study outcomes (see comment above) if referenced in the discussion. The study had a benefit of repeated measures but no analysis leverages this to understand factors associated with changes including awareness (curious that the question administered at baseline did not increase awareness in later surveys for example).

***Table 2 shows that the willingness measures were not significantly different between intervention and control conditions. Their means and standard deviations for the both conditions were very close. Therefore, the intervention effects reported in Table 3 are not only indicating the differences between two conditions at each follow-up point, they are also reflecting the difference of change between two conditions.***

11. The statement "Our finding indicates that addressing specific individual-level barriers, creating chances to build empowerment and advocacy among Black women may facilitate both PrEP initiation and condom use" need more clarification and explanations as the intervention did not actually increase initiation.

***Thank you for your insightful comment regarding the statement above in our discussion section. We appreciate the opportunity to clarify and provide further explanations.***

***You are correct in pointing out that our intervention did not directly increase PrEP initiation rates among the study participants. The primary objective of our intervention was to address individual-level barriers and empower Black women through educational sessions and peer support groups. While we did not observe a statistically significant increase in PrEP initiation rates, our qualitative data (not presented in this paper) and participant feedback suggest that the intervention had a positive impact on increasing awareness, knowledge, and self-efficacy regarding PrEP and condom use. Also, by addressing individual-level barriers, such as lack of knowledge, stigma, and perceived risk, and fostering empowerment and advocacy, our intervention created an environment conducive to facilitating PrEP initiation and consistent condom use. However, we acknowledge that the wording may have been misleading, and we appreciate your feedback to clarify this point. We do believe that our findings suggest that the intervention equipped participants with the necessary knowledge, skills, and confidence to make informed decisions about their sexual health and HIV prevention strategies, including PrEP initiation and condom use.***  ***To address your concern and provide clarity, we propose revising the statement below and have relocated it to the last paragraph of the discussion section. We hope this revision and additional explanation has helped clarify our findings and address your concern.***

"Our findings indicate that addressing specific individual-level barriers and could create opportunities for empowerment and advocacy among Black women can increase their knowledge, self-efficacy, and intentions to initiate PrEP and consistently use condoms for HIV prevention."

12. The lack of actual PrEP uptake deserves more discussion including what health system and other factors are potential contributors and how this is similar or different from other studies of PREP awareness, intent and use. The limitation should include any related to the actual outcome questions used.

***Thank you for this reflection. Reviewer 2 had a similar comment. We added to the following paragraph to the conclusion that acknowledges your point of recognizing the importance of awareness alone, and proposing a well-reasoned, multi-faceted approach. We hope this paragraph effectively addresses your comment and provides a clear path forward for bridging the gap between awareness/interest and actual uptake and sustained use of PrEP.:***

“While our results do not directly address ways to increase awareness, uptake and sustained use of PrEP. increasing awareness and interest of PrEP alone is insufficient to drive behavior change and sustained use. To bridge the gap between awareness/interest and actual uptake and adherence, a multi-pronged approach is typically needed that include ensuring affordable and seamless integration of PrEP into healthcare systems, offering counseling and support to address concerns and encourage adherence, leveraging networks and influential figures for outreach, tailoring messages to cultural backgrounds, exploring innovative delivery methods like telemedicine and home delivery, and continuously monitoring and adjusting strategies. Thus, a comprehensive approach addressing individual, social, and structural factors, coupled with ongoing support, is necessary to bridge the gap between interest and consistent PrEP utilization.”

**(Reviewer 1, Minor-the sentence)**

13. "Further, the sample of participants experienced multiple factors that contributed to their vulnerability to HIV transmission, consideration for the reporting of poverty indicators are heavily prevalent among the sample population, with many reporting food insecurity, homelessness, and substance abuse" is incomplete.

***Thank you for pointing it out. We have decided to restructure the entire sentence for clarity. As a result, it now reads as follows:***

*“Further, the sample of participants experienced multiple factors that contributed to their vulnerability to HIV transmission, including high poverty indicators are prevalent among the sample population, with many reporting food insecurity and homelessness”*

**ASCP-D-23-00120**

**Effectiveness of a culturally tailored HIV intervention in promoting PrEP among Black women who use drugs in community supervision programs in New York City: A randomized clinical trial.

Reviewer(s)' Comments to Author:**

**Response to Reviewer# 2**

**(Reviewer# 2:)** You have uncovered an important dynamic and I look forward to seeing similar analyses with original datasets as well.

**Response to Reviewer #2:**

We thank the reviewer for their kind feedback and encouragement. I really appreciate you taking the time to review our work. Regarding conducting similar analyses with original datasets, that is definitely one of our goals moving forward. As part of the parent study, we also conducted in-depth interviews and focus groups, to gain first-hand accounts from participants which will allow us to better understand their experiences, motivations, and decision-making processes better. Many of these findings are currently under review.

We are excited about these possibilities to build upon the findings from our current work. We are grateful for your insights.

Thank you.

_____________________________________________________________________________________________________________________

**(Reviewer 2, Abstract)**

1. Line 30: I think there may be a typo in the sentence beginning with "Moreover, women involved….." I think authors meant to say: "Moreover, women involved in community supervision programs….."

***This has been corrected***

2. Line 40-41: I would list receipt of HIV testing before describing the 5-session intervention, as the way the sentence is laid out is confusing. For example: "Participants were randomized to either HIV testing plus a 5-session, culturally-tailored…… versus an HIV testing alone (n=180)."

***This has been corrected***

3. Line 42: The word report should be changed to reports.

***This has been corrected***

4. Line 44: Last sentence of the methods should read: "HIV outcomes are reported in a previous paper."

***This has been corrected***

**(Reviewer 2, Introduction)**

5. Several typos. Please re-read carefully and correct.

***Our apologies for the numerous typos. The manuscript has been proofread and edited.***

**(Reviewer 2, Methods)**

6. In the "reasons for exclusion" box in figure 1, I believe bullet point (3) should also include no report of binge drinking.

***We have added no report of binge drinking to Figure 1.***

7. Line 249: I believe there is a typo in this sentence, and/or parentheses are out of place.

***This has been corrected.***

**(Reviewer 2, Results)**

8. In general, I find regression coefficients challenging to interpret and would recommend authors consider other ways they can represent the date on willingness to use PrEP.

***This is a limitation of the study to rely on one single item for measuring willingness with assigned numeric 1-4 to the unlikely-likely responses. We have added this to the limitation section. However, although the coefficients are not easily to be interpreted numerically, the coefficients indicate clearly that the EWORTH group reported higher willingness to use PrEP than the control group. The following has been added to the limitation section:***

“Finally, the regression coefficients is a limitation of the study as we relied on one single item for measuring willingness with assigned numeric 1-4 to the unlikely-likely responses. However, although the coefficients are not easily to be interpreted numerically, the coefficients indicate clearly that the EWORTH group reported higher willingness to use PrEP than the control group.”

**(Reviewer 2, Discussion)**

9. Line 326: There is a typo in the first sentence of the discussion. "who" should be "with"

***We rearranged the beginning of the discussion but have addressed the typo*.**

10. Can authors speak to why they think PrEP uptake was so low in both groups?

***Based on additional qualitative interviews done with a portion of the sample, many of the women have severe mistrust of both PrEP and the medical system. These findings are currently under review at another journal. We also addressed this in the discussion as a barrier to PrEP uptake.***

11. If the E-WORTH intervention was effective in increasing awareness and willingness to use PrEP, why did this not translate into at least slightly higher uptake over the 12 months of follow-up, as shown in table 2. This is briefly mentioned in the limitations / conclusion sections, but I think warrants a larger discussion.

***As mentioned above, based on in-depth interviews conducted post 12-month follow-up the mistrust is the biggest barrier. Many of the women also did not see themselves as being high-risk for HIV, though the results on their HIV risk behavior from baseline and follow up said something different. Perhaps going through the EWORTH intervention provided education and post intervention women practiced safer sexual behavior. We have expanded the discussion on this.***

12. This is not a criticism of the study but just a general comment, that I feel like there are several studies that look at ways to increase awareness and interest in PrEP, but it's not clear that this actually translates into higher uptake and sustained use of PrEP - how do we bridge this gap?

***Thank you for this reflection. We added to the following paragraph to the conclusion that acknowledges your point of recognizing the importance of awareness alone, and proposing a well-reasoned, multi-faceted approach. We hope this paragraph effectively addresses your comment and provides a clear path forward for bridging the gap between awareness/interest and actual uptake and sustained use of PrEP.:***

“While our results do not directly address ways to increase awareness, uptake and sustained use of PrEP. increasing awareness and interest of PrEP alone is insufficient to drive behavior change and sustained use. To bridge the gap between awareness/interest and actual uptake and adherence, a multi-pronged approach is typically needed that include ensuring affordable and seamless integration of PrEP into healthcare systems, offering counseling and support to address concerns and encourage adherence, leveraging networks and influential figures for outreach, tailoring messages to cultural backgrounds, exploring innovative delivery methods like telemedicine and home delivery, and continuously monitoring and adjusting strategies. Thus, a comprehensive approach addressing individual, social, and structural factors, coupled with ongoing support, is necessary to bridge the gap between interest and consistent PrEP utilization.”
